# The Expression and Regulatory Roles of Long Non-Coding RNAs in Periodontal Ligament Cells: A Systematic Review

**DOI:** 10.3390/biom12020304

**Published:** 2022-02-12

**Authors:** Yifan Lin, Zhongyuan Tang, Lijian Jin, Yanqi Yang

**Affiliations:** 1 Division of Paediatric Dentistry and Orthodontics, Faculty of Dentistry, The University of Hong Kong, Hong Kong, China; yflin@hku.hk (Y.L.); tzy337@connect.hku.hk (Z.T.); 2 Division of Periodontology and Implant Dentistry, Faculty of Dentistry, The University of Hong Kong, Hong Kong, China; ljjin@hku.hk

**Keywords:** long non-coding RNA, periodontal ligament cells, osteogenic differentiation, inflammation, mechanical stress

## Abstract

Periodontal ligament (PDL) cells play a pivotal role in periodontal and bone homeostasis and have promising potential for regenerative medicine and tissue engineering. There is compelling evidence that long non-coding RNAs (lncRNAs) are differentially expressed in PDL cells compared to other cell types and that these lncRNAs are involved in a variety of biological processes. This study systematically reviews the current evidence regarding the expression and regulatory functions of lncRNAs in PDL cells during various biological processes. A systematic search was conducted on PubMed, the Web of Science, Embase, and Google Scholar to include articles published up to 1 July 2021. Original research articles that investigated the expression or regulation of lncRNAs in PDL cells were selected and evaluated for a systematic review. Fifty studies were ultimately included, based on our eligibility criteria. Thirteen of these studies broadly explored the expression profiles of lncRNAs in PDL cells using microarray or RNA sequencing. Nineteen studies investigated the mechanisms by which lncRNAs regulate osteogenic differentiation in PDL cells. The remaining 18 studies investigated the mechanism by which lncRNAs regulate the responses of PDL cells to various stimuli, namely, lipopolysaccharide-induced inflammation, tumor necrosis factor alpha-induced inflammation, mechanical stress, oxidative stress, or hypoxia. We systematically reviewed studies on the expression and regulatory roles of lncRNAs in diverse biological processes in PDL cells, including osteogenic differentiation and cellular responses to inflammation, mechanical stress, and other stimuli. These results provide new insights that may guide the development of lncRNA-based therapeutics for periodontal and bone regeneration.

## 1. Introduction

Periodontitis is a plaque-induced inflammatory oral disease that causes the progressive breakdown of periodontal tissue, and it is one of the leading causes of tooth loss [1,2]. Although conventional therapies can control active periodontal inflammation, they are unable to fully regenerate damaged periodontal tissue. Therefore, recent efforts to treat periodontal diseases have focused on regenerative therapies that can restore the physiological function of teeth by re-building supporting periodontium, including periodontal ligament (PDL), alveolar bone, gingiva, and cementum [3,4].

PDL is a thin layer of fibrous connective tissue, located between the alveolar bone and cementum, that plays a crucial role in the development, functioning, and regeneration of the tooth-supporting apparatus. An early study of PDL found that it had a regenerative capacity and possibly contained a population of multipotent progenitor cells [5]. It has since been established that PDL cells are a heterogeneous cell population consisting of fibroblastic and osteoblastic mesenchymal lineages that include cells at different stages of differentiation and lineage commitment [6,7,8,9,10,11]. PDL stem cells (PDLSCs) were first isolated in 2004 [12] and have been shown to exhibit self-renewal ability and multipotent capacities. Numerous in vitro studies have revealed that PDLSCs can differentiate into various types of cells, including adipocytes, osteoblasts, chondrocytes, neurons, and hepatocytes [12,13,14,15]. Moreover, in vivo models have demonstrated that PDLSCs form cementum- and PDL-like structures after transplantation into surgically created periodontal defects, which suggests that they could be used for the regeneration of periodontal tissues [16,17,18]. A study comparing PDL cells with PDLSCs revealed that PDL cells were similar to PDLSCs in that they have a high proliferative capacity and multipotent differentiation abilities, express mesenchymal surface markers, and can regenerate periodontal tissues in vivo [19]. This study also demonstrated the feasible and safe application of autologous PDL cells for periodontal regenerative treatment in patients diagnosed with periodontitis [19].

RNAs are versatile biomolecules that are either protein-coding (coding) or non-protein-coding (non-coding) RNAs. Non-coding RNAs, which are not translated into proteins, are classified as housekeeping RNAs (e.g., ribosomal RNAs, transfer RNAs, and small nuclear RNAs) and regulatory RNAs [20]. Regulatory RNAs are further classified based on their length into short and long non-coding RNAs (lncRNAs). MicroRNAs (miRNAs) are short non-coding RNAs that are generally 20 to 23 nucleotides in length, and they function as post-transcriptional repressors by binding to messenger RNA (mRNA), which results in the silencing of a specific target gene.

LncRNAs have received much attention in recent years and comprise a large and diverse class of transcribed RNA molecules that are greater than 200 nucleotides in length. It was discovered that these lncRNAs, which were once considered “transcriptional noise” in the genome, may play critical regulatory roles in many biological processes [21]. However, due to their low conservation, high level of alternatively spliced transcripts, and tissue- and development-specific expression, most lncRNAs remain unannotated and are yet to be ascribed any function [10]. LncRNAs typically interact with DNA, RNA, protein molecules, and/or combinations thereof to regulate gene expression at transcriptional and post-transcriptional levels [22].

Studies have indicated that lncRNAs are involved in and may be vital to a variety of diseases associated with aberrant cellular control, including autoimmune, neurological and cardiovascular conditions, and cancers [23,24,25,26]. In recent years, the role of lncRNAs in the regulation of PDL cells has attracted increasing attention. There is substantive evidence that lncRNAs are differentially expressed in PDL cells compared to other cell types and are also differentially expressed during various biological functions [27,28,29,30]. In addition, several lncRNAs, such as maternally expressed gene 3 (MEG3), anti-differentiation non-coding RNA (ANCR), and taurine upregulated gene 1 (TUG1), have been found to regulate the regenerative capacities of PDL cells under normal and inflammatory conditions [31,32,33].

The significant progress made toward elucidating the biogenesis and functions of lncRNAs has afforded ample evidence for their critical roles in many biological pathways. The present study systematically reviews articles on the expression and regulatory roles of lncRNAs in PDL cells during a variety of biological processes.

## 2. Materials and Methods

### 2.1. Search Strategy

A systematic search for studies was conducted in four databases (PubMed, Clarivate-Web of Science, Google Scholar, and Embase) from the date of their respective inception to 1 July 2021. The search terms used were as follows: (“periodontal ligament cell” OR “periodontal ligament stem cell” OR “periodontal ligament fibroblast” OR “PDL cell” OR “PDLSC” OR “PDLC” OR “hPDLSC” (human PDLSC) OR “hPDLC” (human PDLC) OR “hPDL cell” (human PDL cell) AND “long non-coding RNA” OR “long noncoding RNA” OR “lncRNA”.

### 2.2. Selection Criteria

The inclusion criteria were as follows: (1) studies based on cell, human, or animal models; (2) studies related to the expression or regulation of lncRNAs in PDL cells; and (3) studies published in English. The exclusion criterion was as follows: reviews, conference abstracts, or editorials.

### 2.3. Selection of Studies

Titles and abstracts of manuscripts were independently screened in electronic sheets by two reviewers (Y.L. and Z.T.). Titles and abstracts were examined, and duplicate studies were eliminated. If an article’s abstract did not contain sufficient information for an inclusion/exclusion decision to be made, its full text was obtained and carefully inspected. Any inter-examiner disagreement was resolved by discussion. The level of agreement between the two examiners was assessed by determining Cohen’s kappa scores.

### 2.4. Quality Assessment

The quality of selected papers was evaluated using a well-known system (Table S1) described by Wells and Littell [34]. The following eight questions comprised the quality scoring system. (1) Was the study hypothesis/aim/objective clearly described? (2) Were the experimental designs in the study well described? (3) Were the methods and materials in the study well described? (4) Were the time-points of data collection in the study clearly defined? (5) Were the main outcomes of measurements in the study clearly defined? (6) Were the experimental groups comprehensively compared with the control group in the study? (7) Were the results in the study well described? (8) Were the limitations of the study discussed? In answering each question, 1 point was allocated for “yes” and 0 points were allocated for “no.” The sum of scores for each study was calculated independently, and the total possible score was 8. A score of 7 to 8 indicates a study with excellent quality, a score of 5 to 6 indicates a good quality study, a score of 3 to 4 indicates a low-quality study, and a score of 0 to 2 indicates a bad quality study. A detailed evaluation of the scores of selected studies is presented in Table S1.

## 3. Results

### 3.1. Literature Search and Screening of Studies

**Figure 1:**
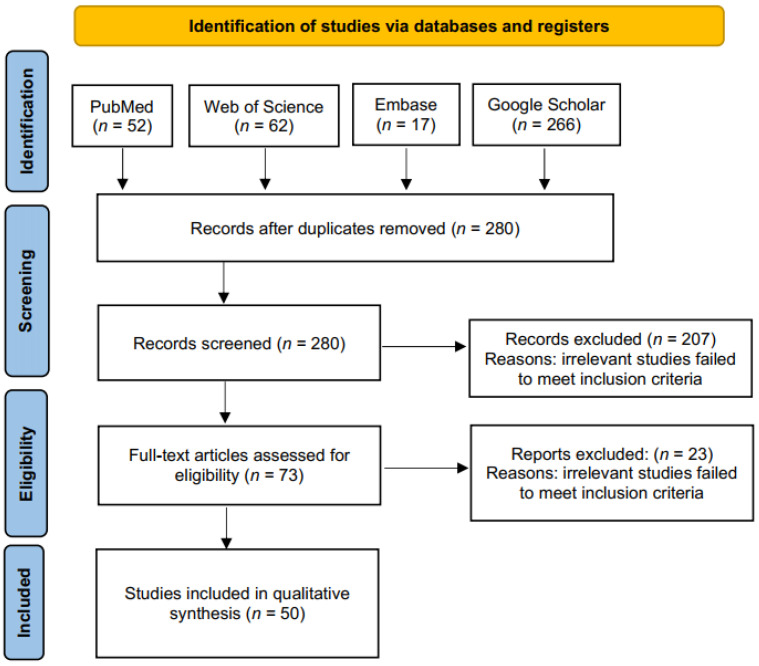
Flow diagram of the process of study selection. Two hundred and eighty studies were retrieved based on the search strategy described in the methodology. After reviewing the titles and abstracts of these studies, 73 studies were obtained. After reviewing the full text of these studies, 50 studies met the criteria for inclusion in this systematic review.

**Figure 2:**
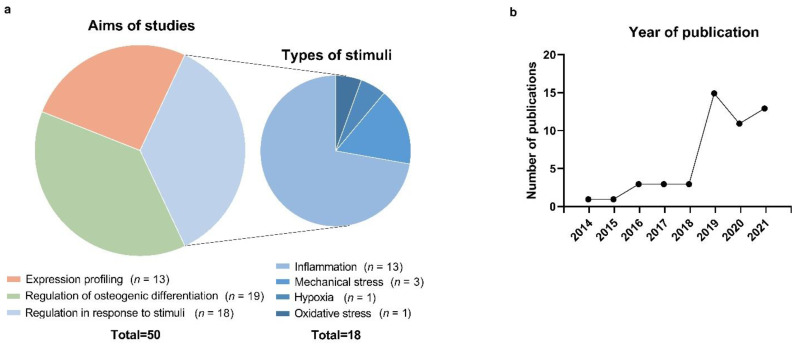
Characteristics of the studies included in the systematic review. (**a**) Aims of the included studies and types of stimuli. (**b**) Distribution of years of publication of included studies.

A flow diagram of study selection is shown in Figure 1. Two hundred and eighty records were obtained by screening titles and abstracts and removing duplicates. After reviewing these titles and abstracts, 73 articles were retrieved for full-text evaluation, and 23 were subsequently excluded for the reasons described in the diagram. The remaining 50 studies were included for further analysis. The kappa score for study selection was 0.939, indicating that there was an excellent level of agreement between the reviewers. All studies were published between 2014 and 2021, and their characteristics are summarized in Figure 2.

### 3.2. Studies on lncRNA Expression Profiling in PDL Cells

In total, 13 of the 50 studies broadly explored the expression profiling of PDL cells using microarray or RNA sequencing (RNA-seq) (Table 1). These studies investigated the lncRNA expression profiles of PDL cells subjected to osteogenic induction [28,29,30,35,36] and mechanical stress [37,38,39,40,41]; some compared PDL cells’ expression profiles with those of other cell types, including bone marrow stem cells (BMSCs) [27], gingival mesenchymal stem cells (GMSCs) [42], and dental follicle cells (DFCs) [43].

### 3.3. Studies on lncRNAs Involved in the Osteogenic Differentiation of PDL Cells

Table 2 presents 19 studies that investigated the mechanism by which lncRNAs regulate the osteogenic differentiation of PDL cells. Fifteen of these studies investigated PDL cells from healthy individuals (henceforth denoted as H-PDL cells or H-PDLSCs) [31,32,33,44,45,46,47,48,49,50,51,52,53,54,55,56,57,58,59], whereas four of these studies isolated PDL cells from patients with periodontitis (henceforth denoted as P-PDL cells or P-PDLSCs) to determine the regulatory role of lncRNAs in osteogenesis under inflammatory conditions [33,45,50,58].

### 3.4. Studies on lncRNAs in PDL Cells Subjected to Inflammation, Mechanical Stress, and Other Stimuli

Table 3 presents 18 studies that investigated the role of lncRNAs in regulating cellular processes in PDL cells in the presence or absence of stimuli, including studies that compared H-PDL and P-PDL cells [60,61,62,63,64,65]. These studies also explored the role of lncRNAs in lipopolysaccharide (LPS)-induced inflammation [66,67,68,69,70,71], tumor necrosis factor-alpha (TNF-α)-induced inflammation [72], mechanical stress [73,74,75], hypoxia [76], and oxidative stress [77]. To review the outcomes of lncRNA involvement, cell proliferation, apoptosis, inflammatory responses, autophagy, migration, and root resorption were examined.

## 4. Discussion

This study systematically reviewed studies exploring the expression of lncRNAs and their role in the regulation of a variety of biological activities in PDL cells, such as osteogenesis and cell response to inflammation and mechanical stress. LncRNAs regulate gene expression at transcriptional and post-transcriptional levels. At the transcriptional level, lncRNAs may directly bind to DNA or act on transcriptional complexes, resulting in cis or trans gene activation or silencing [78,79]. LncRNAs can recognize and bind to complementary RNA sequences, which enables highly specific interactions that can regulate various post-transcriptional processes, such as mRNA splicing, transport, translation, and stabilization, thereby affecting various biological processes [78,80]. LncRNAs can also specifically recruit and integrate with RNA binding proteins (RBPs) to regulate their biological functions, thereby affecting the expression of downstream genes [81]. In addition to regulating mRNAs via independent mechanisms, lncRNAs can act as competing endogenous RNAs (ceRNAs) by competitively binding to miRNAs via miRNA response elements. This binding attenuates the ability of miRNAs to downregulate mRNA expression and thus indirectly regulates mRNA expression [82]. LncRNA-mediated ceRNA interactions have been identified in various cancers and inflammatory diseases, including periodontitis [83,84,85].

### 4.1. Studies on lncRNA Expression Profiling in PDL Cells

In the 13 studies that explored lncRNA expression profiles in PDL cells, 6 used microarray analysis and 7 used RNA-seq methods [27,28,29,30,35,36,37,38,39,40,41,42,43]. Recent developments in RNA-seq techniques offer enormous potential for transcriptome characterization as they are reliable tools for elucidating genetic and metabolic pathways involved in biological processes. RNA-seq provides more comprehensive information about the characteristics of transcripts as this information is not limited to the known genes represented on a microarray and novel transcription variants can be detected via alternative splicing [86].

Three of these thieteen studies compared lncRNA expression profiles in PDLSCs with the lncRNA expression profiles of other cell types, such as BMSCs, GMSCs, and DFCs [27,42,43]. Moreover, 5 of these 13 studies examined the lncRNA expression profiles of PDLSCs under osteogenic induction [28,29,30,35,36]. The results varied, showing differently up- and downregulated lncRNAs during the osteogenic differentiation process. These variations may be attributable to differences between samples and periods of induction. For example, Qu et al. [35] and Zhang et al. [29] examined three osteogenic-induced and three non-induced samples, whereas some authors did not mention the number of samples tested, and the period of osteogenic induction varied widely (3, 5, 7, or 14 days) between studies. It has been suggested that aging can affect the characteristics of the regenerative potentials of dental-derived stem cells [87,88]. Moreover, differences in library preparation, sequencing techniques, and methods of analysis may also have led to the variations in the results. Five of the thirteen studies explored the lncRNA profile of PDL cells subjected to mechanical stress [37,38,39,40,41]. Three of these studies applied tensile force on cells (10% or 12% equibiaxial strain) [37,39,40] and one applied compressive force on cells (2 g/cm^2^) for 12 h [38]. After microarray or RNA-seq, most studies performed only PCR to validate the expression of several genes. Further in-depth studies are warranted to explore the regulation of the identified lncRNAs.

### 4.2. Studies on lncRNAs Involved in the Osteogenic Differentiation of PDL Cells

PDL cells are expected to play an important role in the clinical application of periodontal tissue regeneration as they offer new solutions for the treatment of periodontal diseases [19]. Studies have significantly expanded our knowledge of the potential regulatory role of lncRNAs in the osteogenic differentiation of PDL cells. Nineteen of the included studies explored the mechanism by which lncRNAs regulate the osteogenic differentiation of PDL cells [31,32,33,44,45,46,47,48,49,50,51,52,53,54,55,56,57,58,59]. The lncRNAs TUG1, prostate cancer-associated transcript 1 (PCAT1), X-inactive specific transcript (XIST), Fer-1-like family member 4 (FER1L4), growth arrest-specific transcript 5 (GAS5), Prader Willi/Angelman region RNA 6 (PWAR6), osteogenesis impairment-related lncRNA of PDLSCs from periodontitis patients (lncRNA-POIR), Twist1, and antisense non-coding RNA in the inhibitor of cyclin-dependent kinase 4 (INK4) locus (ANRIL) have been reported to enhance osteogenic differentiation of PDL cells [32,45,48,50,51,52,55,56,58,59]. Whereas the lncRNAs ANCR, small molecule RNA host gene 1 (SNHG1), differentiation antagonizing non-coding RNA (DANCR), hypoxia-inducible factor 1 alpha-antisense RNA 2 (HIF1A-AS2), exportin5 (XPO5), HOX transcript antisense RNA (HOTAIR), and homeobox A (HOXA) transcript at the distal tip (HOTTIP) have been reported to negatively correlate with the osteogenic differentiation of PDL cells [31,44,46,47,53,54,57].

TUG1 was initially identified as an important gene in retinal development and the formation of photoreceptors [89]; later, it was reported to be abnormally expressed during tumorigenesis [90]. It was observed that TUG1 can bind to lin-28 homolog A, an RBP, thereby promoting the expression of osteogenesis-related markers and the osteogenic differentiation of PDLSCs [32]. Wu et al. reported a post-transcriptional regulatory mechanism by which TUG1 enhanced the osteogenic differentiation of PDLSCs: TUG1 sponges microRNA-222-3p, which promotes osteogenic differentiation by upregulating Smad 2/7, which are the main signal transducers for receptors of transforming growth factor beta. The knockdown of TUG1 or overexpression of microRNA-222-3p inhibited this upregulation [55]. MEG3, initially known as a tumor suppressor, is another lncRNA that has received much attention due to its association with the osteogenic differentiation of MSCs, DFCs, and PDL cells [33,49,91,92]. In PDL cells, MEG3 attenuates bone morphogenetic protein 2 (BMP2) expression by competing with BMP2 for binding to the RBP heterogeneous nuclear ribonucleoprotein I [49]. Furthermore, four studies have investigated the regulatory role of lncRNAs in the osteogenesis of P-PDL cells isolated from the extracted teeth of patients with periodontitis [33,45,50,58]. P-PDL cells were first isolated in 2010 and have since attracted much attention [93]. However, P-PDL cells have been shown to have less osteogenic differentiation potential than H-PDL cells [94,95,96,97]. Wang et al. used microarray analysis to identify a novel lncRNA, lncRNA-POIR, which is differentially expressed in P-PDLSCs. They found that LncRNA-POIR regulates Forkhead box O (FOXO)1 by sponging miR-182 and, thus, inhibits the canonical Wnt pathway and promotes osteogenesis [45].

### 4.3. Studies on lncRNAs in PDL Cells Subjected to Inflammation, Mechanical Stress, and Other Stimuli

Inflammation-stimulating factors released by bacteria, such as LPS and TNF-α, activate the immune response in PDL cells, thereby aggravating the destruction of alveolar bone. Among the 18 studies that investigated the regulatory role of lncRNAs in PDL cells in response to inflammation and other stimuli, 6 studies compared the inflammatory responses of H-PDL and P-PDL cells [66,67,68,69,70,71], 7 studies stimulated PDL cells with LPS or/and TNF-α to mimic periodontal inflammation [66,68,69,70,71,72,74], 3 studies stimulated PDL cells with mechanical stress [73,74,75], 1 study subjected PDL cells to hypoxia [76], and 1 study subjected cells to oxidative stress [77]. Under these stimuli, the biological activities of PDL cells, including cell proliferation, apoptosis, inflammatory responses, osteogenic differentiation, and autophagy, were explored.

LPS is an endotoxin and a major component of the cell membranes of Gram-negative bacteria, such as *Porphyromonas gingivalis* and *Escherichia coli*, where it performs various biological activities. It is mediated by the toll-like receptors (TLR) 2, and TLR4 and triggers cytokine-mediated immune-inflammatory responses in the host, which results in the release of a wide range of pro-inflammatory cytokines. Several lncRNAs, including TUG1, MEG3, metastasis-associated lung adenocarcinoma transcript 1 (MALAT1), FGD5-antisense RNA 1 (FGD5-AS1), and LINC01126, have been reported to modulate the inflammatory response of PDL cells to LPS challenge. Huang et al. and Han et al. reported that the expression of TUG1 is decreased in PDL cells upon LPS challenge, but they ascribed this to different regulatory mechanisms [67,71]. Han et al. reported that TUG1 competes with miR-132 to promote the proliferation and inhibit the apoptosis of PDL cells under inflammatory stimuli [67]. More recently, Huang et al. suggested that TUG1 is a sponge of miR-498, which allows it to regulate the expression of RAR-related orphan receptor A and attenuate LPS-induced activation of the Wnt/beta-catenin pathway [71].

There has been extensive research on the regulatory mechanisms of orthodontic tooth movement. PDL cells subjected to mechanical stress are widely used to mimic in vivo conditions. Three lncRNAs, DANCR, MIR31 host gene (MIR31HG), and FER1L4, have been investigated for their role in the regulation of compressive force-induced biological activities in PDL cells [73,74,75]. It was suggested that the knockdown of DANCR inhibits the osteoclast formation and root resorption that is induced by compressive force via miR-34a-5p/jagged1 [73]. In addition, lncRNAs also regulate force-induced autophagy in PDL cells. For example, FER1L4 mediates compression-induced autophagy via the AKT/FOXO3 signaling pathway [75]. Notably, these studies have focused only on the effects of compressive stress on the regulation of lncRNAs in PDL cells; there have been no investigations on the effects of other types of stress loadings, such as tensile or shear forces, on the regulation of lncRNAs in PDL cells.

### 4.4. Future Perspectives

Non-coding RNAs possess critical biological functions that were initially discovered in cancer research and then in stem cell studies, and an increasing number of lncRNAs have been discovered in the field of regenerative medicine. With the rapid development of high-throughput sequencing, it is critical to screen diverse lncRNAs and further investigate their roles in various biological functions. With more in-depth research, lncRNAs and their target genes may be identified as possible therapeutic targets in clinically relevant diseases. This review summarizes current research on lncRNAs in PDL cells, with a focus on the expression profile of lncRNAs, their regulation of osteogenic differentiation and the effect upon stimulations. However, most of these recent developments are still in the in vitro stage, and clinical application remains a challenge.

## 5. Conclusions

PDL cells have significant potential for use in the clinical application of periodontal and bone regeneration. This study systematically reviewed studies exploring the expression and regulatory roles of lncRNAs in the diverse biological processes of PDL cells, such as osteogenic differentiation and cellular responses to inflammation, mechanical stress, and other stimuli. However, most of these studies were focused on in vitro analyses; more in vivo investigations are required in this promising translational field.

## Figures and Tables

**Table 1:** Studies on expression profiling of lncRNAs in PDLSCs.

Study	Samples and Stimulation	Differential Expression of lncRNAs in PDL Cells	qPCR Validation
[27]	3 PDLSC and 3 BMSC samples	457↑ and 513↓ lncRNAs in PDLSCs	↑: NR045555, NR027621, NR03365; ↓: NR037182, NR037595, XR111050 (in PDLSCs)
[28]	osteogenic-induced and non-induced PDLSC samples	777↑ and ↓ lncRNAs in induced PDLSCs (|fold change| ≥ 2 and *p* < 0.05)	↑: TCONS_00019601, TCONS_00227764, TCONS_00254538, TCONS_00198784, TCONS_00136898; ↓: TCONS_00085268, TCONS_00125934, TCONS_00115113
[29]	3 osteogenic-induced samples, 3 osteogenic- and TNFα-stimulated samples, and 3 non-induced/stimulated samples	214↑ and 193↓ lncRNAs in osteogenic-induced PDLSCs; 149↑ and 169↓ lncRNAs in TNFα- and osteogenic-induced PDLSCs compared to non-induced PDLSCs (log2 fold-change ≥ 1 and adjusted *p* ≤ 0.05).	↑: LINC-PDE10A-1, GK-AS-1; ↓: ZNF385D-AS-1, SGOL1-AS-1
[30]	osteogenic-induced and non-induced PDLSC samples	10, 36 and 69↑ and 44, 11 and 70↓ lncRNAs after 3 days, 7 days, and 14 days of osteogenic induction, respectively (fold-change ≥ 2 and adjusted *p* < 0.05).	↑: MEG8, MIR22HG
[35]	3 osteogenic-induced and 3 non-induced PDLSC samples from 15 individuals	994↑ and 1177↓ lncRNAs in induced PDLSCs (|fold change| ≥ 2 and *p* < 0.05)	↑: AC078851.1, RP11-45A16.4, XLOC_002932, RP4-613B23.1, RP11305L7.6
[36]	osteogenic-induced and non-induced exosomes derived from PDLSCs	118 (70↑ and 48↓) and 43 (24↑ and 19↓) lncRNAs after 5 or 7 days of osteogenic induction, respectively (*p* < 0.05 and log2 fold-change > 1).	SNHG5, LOC100130992, and ATP6V1B1-AS1: no significant difference.
[37]	3 orthodontic force-induced and 3 non-induced PDL samples	DLEU2↑ and DNAJC3-AS1↓ in induced PDL samples (*p* ≤ 0.05)	/
[38]	compressive force-induced and non-induced PDLSCs	72↑ and 18↓ lncRNAs in compression-induced PDLSCs (adjusted *p* < 0.05 and fold-change > 1.5)	↑: FER1L4, HIF1A-AS2, MIAT, NEAT1, ADAMTS9-AS2, LUCAT1; ↓: MIR31HG and DHFRP1
[39]	5 tension-induced and 5 non-induced PDL cell samples	107↑ and 88↓ lncRNAs in tension-induced-PDL cells (adjusted *p* < 0.05)	↑: MIR22HG, CYTOR, SNHG3
[40]	3 H-PDLSC and 3 P-PDLSC samples	ENST00000411904 the most ↑ lncRNA in strained H-PDLSCs; lncRNA-XIST and ENST0000051750 the most ↑ and ↓ lncRNAs in strained P-PDLSCs, respectively.	↓: TCONS_00008604, ENST00000428781, uc004arq.1, XIST
[41]	tensile force-induced and non-induced PDLSC	799↑ and 540↓ lncRNAs in tension-induced PDLSC (*p* < 0.05, fold-change > 2)	↑: TCONS_00103186, TCONS_00114231, TCONS_00015104, TCONS_00046925, TCONS_00022234; ↓: TCONS_00195572.
[42]	3 PDLSC and 3 GMSC samples	735↑ and 1427↓ lncRNAs in PDLSCs (fold-change ≥ 1.2).	↑: NR_038849, TCONS_l2_00010766-XLOC_l2_005781, ENST00000450854; ↓: n341766, n337408, n385309 (in PDLSCs)
[43]	PDL cell and DFC samples from 4 individuals	385↑ and 460↓ lncRNAs in PDL cells	↑: NR_033917, NR_038367, NR_026861; ↓NR_102703, NR_110162, ENST00000430859 (in PDL cells)

↑: increased, ↓: decreased, bone-marrow stem cell (BMSC), cytoskeleton regulator RNA (CYTOR), dental follicle cells (DFC), gingival mesenchymal stem cell (GMSC), hypoxia-inducible factor 1 alpha-antisense RNA 2 (HIF1A-AS2), MIR31 host gene (MIR31HG), periodontal ligament (PDL), periodontal ligament stem cell (PDLSC), quantitative polymerase chain reaction (PCR), X-inactive-specific transcript (XIST).

**Table 2:** Studies on the regulatory mechanisms of lncRNAs in PDLSCs during osteogenic differentiation.

Study	lncRNAs	Increased (↑) or Decreased (↓) Expression in PDL Cells upon Stimulation	Effect on Osteogenesis	Effect on the Associated Signaling Pathway
[31,44,47]	ANCR	↓ upon osteogenic induction	↓	inhibition of miR-758, which upregulates Notch2- Wnt/β-catenin; inhibition of the Wnt/β-catenin signaling pathway
[54]	DANCR	↓ upon osteogenic induction	↓	/
[52]	FER1L4	↑ upon osteogenic induction	↑	inhibition of miR-874-3p, which regulates the VEGFA axis
[56]	GAS5	↑ upon osteogenic induction	↑	upregulation of GDF5, which decreases the phosphorylation of p38/JNK
[46]	HIF1A-AS2	↑ upon hypoxia	↓	inhibition of HIF-1α
[58]	LncRNA ANRIL	↓ in P-PDLSCs	↑	inhibition of miR-7-5p, which regulates the IGF-1R axis
[45]	LncRNA-POIR	↓ in P-PDLSCs, ↑ upon osteogenic induction	↑	inhibition of miR-182, which downregulates the FoxO1/canonical Wnt pathway
[50]	LncRNA-TWIST1	↓ in P-PDLSCs, ↑ upon osteogenic induction	↑	activation of the Wnt/β-catenin signaling pathway
[33]	MEG3	↓ in P-PDLSCs, ↑ upon osteogenic induction in PDLSCs	↑	inhibition of miR-27a-3p, which regulates the IGF1 axis-regulated PI3K/AKT signaling pathway
[49]	MEG3	↓ upon osteogenic induction	↓	competes with BMP2 mRNA for RBP hnRNPI
[48]	PCAT1	↑ upon osteogenic induction	↑	inhibition of miR-106a-5p, which regulates the BMP2 and E2F5 feed-forward regulatory network
[59]	PWAR6	↑ upon osteogenic induction	↑	inhibition of miR-106a-5, which regulates the BMP2 axis
[53]	SNHG1	↓ upon osteogenic induction	↓	activation of H3K27 trimethylation of the KLF2 promoter
[32,55]	TUG1	↑ upon osteogenic induction	↑	inhibition of miR-222-3p, which downregulates the Smad2/7 ceRNA regulatory network; binding the RNA-binding protein (RBP) Lin28A
[51]	XIST	↑ upon osteogenic induction	↑	inhibition of the miR-214-3p axis
[57]	XPO5, HOTAIR, HOTTIP	↓ in PDLSCs with high osteogenic potentials	↓	/

↑: increased, ↓: decreased, anti-differentiation non-coding RNA (ANCR), antisense non-coding RNA in the INK4 locus (ANRIL), bone morphogenetic protein 2 (BMP2), differentiation antagonizing non-coding RNA (DANCR), exportin 5 (XPO5), Fer-1-like family member 4 (FER1L4), forkhead box protein O1 (FOXO1), growth arrest-specific transcript 5 (GAS5), heterogeneous nuclear ribonucleoprotein I (hnRNPI), histone H3 lysine 27 (H3K27), HOX transcript antisense RNA (HOTAIR), HOXA transcript at the distal tip (HOTTIP), hypoxia-inducible factor 1 alpha-antisense RNA 2 (HIF1A-AS2), insulin-like growth factor 1 (IGF1), Kruppel-like factor 2 (KLF2), Lin-28 homolog A (Lin28A), maternally expressed gene 3 (MEG3), osteogenesis impairment-related lncRNA of PDLSCs from periodontitis patients (lncRNA-POIR), phosphatidylinositol 3-kinase (PI3K), Prader Willi/Angelman region RNA 6 (PWAR6), prostate cancer-associated transcript 1 (PCAT1), protein kinase B (AKT), small molecule RNA host gene 1 (SNHG1), taurine-upregulated gene 1 (TUG1), X-inactive-specific transcript (XIST).

**Table 3:** Studies on the regulatory mechanisms of lncRNAs in PDL cells in response to inflammation, mechanical loading, and other stimuli.

Study	lncRNAs	Increased (↑) or Decreased (↓) Expression in PDLSCs upon Stimulation	Effect on PDLSCs upon Stimulation	Regulatory Mechanism	Associated Signaling Pathways or Biomarkers
[73]	DANCR	↑ in H-PDL cells under compressive force	↑ root resorption	miR-34a-5p/jagged1	silences DANCR, downregulates number of TRAP-positive osteoclasts and the expression of RANKL.
[65]	DCST1-AS1	↓ in P-PDL cells	↓ proliferation	miR-21/PLAP-1	↓ CDK4, CDK6, CCND1; ↑ PLAP-1
[75]	FER1L4	↑ in H-PDLSC under compressive force	↑ autophagy	AKT/FOXO3 signaling pathway	↑ LC3 II/I, Beclin 1, autophagosomes, autolysosomes; ↓ p-FOXO3, p-AKT
[66]	FGD5-AS1	↓ in P-PDL cells and LPS-induced H-PDL cells	↑ proliferation; ↓ apoptosis	miR-142-3p/SOCS6/NF-κB pathway	↓ p/t-p65, BAX/Bcl-2, cleaved/pro-caspase-3, cleaved/pro-caspase-9, TNF-α, IL-6, IL-1β, and IL-8; ↑ p/t-IκBα
[72]	H19	↑ in TNF-α and LPS-induced H-PDL cells	↑ autophagy	PI3K/AKT signaling pathway.	↑ Beclin-1, LC3 II/I, TNF-α, and IL-6; ↓ p-AKT
[77]	JHDM1D-AS1	↓ in H_2_O_2_-induced H-PDLSC	↓ apoptosis	DNAJC10/p-eIF2α/Bcl-2 regulatory axis	↓ cleaved-caspase 3, cleaved-caspase 9, BAK, ROS, DNAJC10; ↑ p-PERK, p-eIF2α, Bcl-2/BAX
[69]	LINC01126	↑ in LPS-induced H-PDL cells	↑ inflammation; ↓migration	MEK/ERK signaling pathway	↓ p/t-MEK and p/t-ERK.
[76]	LINC01126	↑ in hypoxia-induced H-PDL cells	↑ apoptosis, inflammation;↓ proliferation	miR-518a-5p/HIF-1α/MAPK pathway	↑ p38, ERK1/2, JNK, IL-1β, IL-6, IL-8, TNF-α.
[64]	Linc-RAM	↓ in P-PDLSC	↑ proliferation	inhibits the effect of overexpression of FGF2 on proliferation	/
[63]	MAFG-AS1	↓ in P-PDLSC	↑ inflammation; ↓ proliferation	miR-146a/TLR4 axis	↑ TLR4
[60]	MALAT1	↑ in P-PDLSC	↑ proliferation	FGF2 axis	↑ FGF2
[70]	MALAT1	↑ in LPS-induced H-PDL cells	↑ apoptosis, inflammation ↓ proliferation	miR-769-5p/HIF3A axis	↑ IL-6, IL-1β, TNF-α, BAX, and caspase-3; ↑ Bcl-2.
[68]	MEG3	↓ in P-PDL cells and LPS-induced H-PDL cells	↑ proliferation;↓ apoptosis, inflammation	miR-143-3p AKT/IKK pathway	↓ p-AKT/AKT, p-IKK/IKK, p-p65, IL-6, IL-18, IL-1β, TNF-α.
[74]	MIR31HG	↓ in H-PDLSC under compressive force	↑ proliferation	DNMT1 and DNMT3B inhibited expression of MIR31HG	silences MIR31HG, inhibits cell viability.
[62]	MORT	↓ in P-PDLSC	↓ proliferation		inhibits cell viability
[61]	PTCSC3	↓in P-PDL cells	↓ proliferation	TLR4	↓ TLR4
[67,71]	TUG1	↓ in P-PDL cells and LPS-induced H-PDL cells	↑ proliferation;↓ apoptosis, inflammation	miR-498/RORA axis and Wnt/β-catenin signaling pathway; miR-132 axis	↓ β-catenin, p/t-GSK-3β, p21, TNF-α, IL-1β, IL-6, and IL-8; ↑ CDK2 and cyclin D1.

↑: increased, ↓: decreased, differentiation antagonizing noncoding RNA (DANCR), DNA methyltransferase 1 (DNMT1), DNA methyltransferase 3B (DNMT3B), domain containing 1-antisense (DCST1-AS1), eukaryotic translation initiation factor 2 subunit alpha (eIF2α), Fer-1-like family member 4 (FER1L4), FGD5-antisense RNA 1 (FGD5-AS1), fibroblast growth factor 2 (FGF2), hypoxia-inducible factor 3 alpha (HIF3A), Linc-RNA activator of myogenesis (Linc-RAM), lipopolysaccharide (LPS), MAF bZIP transcription factor G antisense RNA 1 (MAFG-AS1), maternally expressed gene 3 (MEG3), metastasis-associated lung adenocarcinoma transcript 1 (MALAT1), MIR31 host gene (MIR31HG), mortal obligate RNA transcript (MORT), papillary thyroid carcinoma susceptibility candidate 3 (PTCSC3), periodontal ligament-associated protein-1 (PLAP-1), protein kinase-like endoplasmic reticulum kinase (PERK), protein kinase B (AKT), taurine-upregulated gene 1 (TUG1), toll-like receptors (TLR), tumor necrosis factor alpha (TNF-α).

## Data Availability

Not applicable.

## Supplementary Materials

The following supporting information can be downloaded at: https://www.mdpi.com/article/10.3390/biom12020304/s1, Table S1. Quality Assessment.
