# Supplementary material for: The Expression and Regulatory Roles of Long Non-Coding RNAs in Periodontal Ligament Cells: A Systematic Review
Source: Biomolecules. 2022 Feb 12;12(2):304. doi: 10.3390/biom12020304 (PMC8869287; doi:10.3390/biom12020304)
Supplement: Supplementary file 1 [file biomolecules-12-00304-s001.zip › Table S1.pdf]

Section and topic	No.	Quality criteria	Yes	No
Title/keywords/introduction	1	Was the study hypothesis/aim/objective clearly described?		
Method	2	Were the experimental designs in the study well described?		
	3	Were the methods and materials in the study well described?		
	4	Were the time-points of data collection in the study clearly defined?		
	5	Were the main outcomes of measurements in the study clearly defined?		
	6	Were the experimental groups comprehensively compared with the control group in the study?		
Discussion	7	Were the results in the study well described?		
	8	Were the limitations of the study discussed?		

Study		1	2	3	4	5	6	7	8	Quality score
Assis et al.	[57]	Yes	Yes	Yes	Yes	No	Yes	Yes	No	6
Bian et al.	[58]	Yes	Yes	Yes	Yes	Yes	Yes	Yes	Yes	8
Chen et al.	[46]	Yes	No	Yes	Yes	No	Yes	Yes	No	5
Chen et al.	[66]	Yes	Yes	Yes	No	Yes	Yes	Yes	No	6
Chen et al.	[60]	No	Yes	Yes	Yes	Yes	No	Yes	No	5
Chen et al.	[70]	Yes	Yes	No	No	Yes	No	Yes	No	6
Dong et al.	[27]	Yes	Yes	Yes	No	Yes	Yes	Yes	No	6
Dong et al.	[68]	Yes	No	Yes	Yes	Yes	Yes	No	No	5
Feng et al.	[51]	Yes	No	Yes	Yes	No	Yes	No	No	4
Gu et al.	[28]	Yes	Yes	Yes	No	Yes	Yes	No	No	5
Guo et al.	[72]	Yes	Yes	Yes	Yes	Yes	Yes	Yes	Yes	8
Guo et al.	[37]	Yes	Yes	No	No	Yes	Yes	Yes	No	5
Han et al.	[67]	Yes	Yes	Yes	No	No	Yes	Yes	No	5
Han et al.	[74]	No	Yes	Yes	Yes	Yes	Yes	No	No	5
He et al.	[32]	Yes	Yes	Yes	Yes	Yes	Yes	Yes	No	7
Huang et al.	[71]	Yes	Yes	Yes	Yes	Yes	Yes	Yes	No	7
Huang et al.	[52]	Yes	Yes	Yes	Yes	Yes	Yes	Yes	No	7
Huang et al.	[75]	Yes	Yes	Yes	Yes	Yes	Yes	Yes	Yes	8
Huang et al.	[38]	No	Yes	Yes	Yes	Yes	Yes	No	Yes	6
Jia et al.	[48]	Yes	Yes	Yes	Yes	Yes	Yes	Yes	No	7
Jia et al.	[42]	Yes	No	Yes	Yes	Yes	Yes	Yes	No	6
Jia et al.	[44]	Yes	Yes	Yes	Yes	No	Yes	Yes	No	6
Jia et al.	[31]	Yes	Yes	Yes	Yes	Yes	Yes	Yes	Yes	8
Li et al.	[53]	Yes	Yes	Yes	Yes	Yes	Yes	Yes	No	7
Lin et al.	[39]	Yes	Yes	Yes	Yes	Yes	Yes	Yes	Yes	8
Liu et al.	[40]	Yes	Yes	Yes	Yes	Yes	No	Yes	No	6
Liu et al.	[61]	Yes	No	Yes	No	Yes	No	Yes	No	4
Liu et al.	[33]	Yes	Yes	Yes	Yes	Yes	Yes	No	No	6
Liu et al.	[49]	Yes	Yes	Yes	Yes	Yes	Yes	Yes	No	7
Peng et al.	[47]	Yes	Yes	No	Yes	Yes	Yes	Yes	No	6
Qu et al.	[35]	Yes	Yes	Yes	Yes	Yes	Yes	Yes	No	7
Shi et al.	[77]	Yes	Yes	Yes	Yes	Yes	Yes	No	No	6
Wang et al.	[41]	Yes	Yes	No	Yes	Yes	Yes	Yes	No	6
Wang et al.	[45]	Yes	Yes	Yes	Yes	Yes	Yes	Yes	No	7
Wang et al.	[65]	Yes	Yes	Yes	Yes	Yes	Yes	Yes	Yes	8
Wang et al.	[62]	Yes	Yes	No	Yes	No	Yes	Yes	No	5
Wang et al.	[54]	Yes	Yes	Yes	Yes	No	Yes	No	Yes	6
Wang et al.	[63]	No	No	No	Yes	Yes	Yes	Yes	No	4
Wu et al.	[55]	Yes	Yes	Yes	Yes	Yes	Yes	Yes	No	7
Wu et al.	[43]	Yes	Yes	No	Yes	Yes	Yes	Yes	No	6
Wu et al.	[64]	Yes	Yes	Yes	Yes	No	Yes	Yes	No	6
Xiang et al.	[59]	Yes	Yes	Yes	Yes	Yes	Yes	Yes	No	7
Xie et al.	[36]	Yes	Yes	Yes	Yes	Yes	Yes	No	No	6
Xu et al.	[50]	Yes	Yes	Yes	Yes	Yes	Yes	Yes	No	7
Yang et al.	[56]	Yes	No	Yes	No	Yes	Yes	Yes	Yes	6
Zhang et al.	[29]	Yes	Yes	Yes	Yes	Yes	Yes	No	No	6
Zhang et al.	[73]	Yes	Yes	Yes	Yes	Yes	Yes	Yes	Yes	8
Zheng et al.	[30]	Yes	Yes	Yes	Yes	Yes	No	Yes	No	6
Zhou et al.	[76]	No	No	Yes	Yes	Yes	Yes	Yes	No	5
Zhu et al.	[69]	Yes	Yes	Yes	Yes	Yes	Yes	Yes	No	7
